# Association of the duration of post-thaw culture with clinical outcome after vitrified-warmed day 3 embryo transfer in 10,464 cycles: A retrospective cohort study

**DOI:** 10.1097/MD.0000000000021660

**Published:** 2020-08-14

**Authors:** Haiyan Zhu, Wen Xu, Xiaoying Jin, Yamei Xue, Xiaomei Tong, Songying Zhang

**Affiliations:** Assisted Reproduction Unit, Department of Obstetrics and Gynecology, Sir Run Run Shaw Hospital, School of Medicine, Zhejiang University, Key Laboratory of Reproductive Dysfunction Management of Zhejiang Province, Hangzhou, China.

**Keywords:** implantation rate, live birth rate, post-thaw culture duration, pregnancy rate, vitrified-warmed embryo transfer

## Abstract

This study aimed to investigate the effect of the duration of embryo culture on clinical outcome in vitrified-warmed cycles.

This retrospective cohort study enrolled 10,464 infertile patients, with a total of 18,843 vitrified-warmed day 3 embryos from 2012 to 2017 at a single center. The patients were divided into 2 groups: 9470 cycles in the short-term culture group (0.5–8 hours of post-thaw culture) and 994 cycles in the 48 to 72 hours culture group. The independent effect of the following variables on clinical outcomes was determined: duration of post-thaw culture, maternal age, transferred embryos, embryo quality, and endometrial thickness.

We found that the pregnancy rate was positively associated with the post-thaw culture time. Ordinary least square regression analyses showed that the duration of post-thaw culture was positively associated with implantation and live birth rates overall. However, the implantation and live birth rates were not significantly associated with the post-thaw culture time in the short-term culture group. Additionally, maternal age and the number of transferred embryos were independent predictors of the implantation and live birth rates. Moreover, the duration of post-thaw culture did not affect live birth weight.

These results indicated that the pregnancy rate is positively associated with the duration of post-thaw culture. Therefore, under the condition of not affecting work shifts, properly prolonging the duration of post-thaw culture to improve the outcome of frozen-thawed embryo transfer should be considered.

## 1 Introduction

Refinement in cryopreservation techniques and improvement in the pregnancy rate after frozen embryo transfer have facilitated the increasing use of frozen-thawed embryo transfer (FET) globally.^[1,2]^ FET reduces the risk of ovarian hyperstimulation syndrome,^[3]^ without facing the possible adverse effects of supraphysiological hormonal levels over endometrial receptivity and it avoids embryo–endometrial asynchrony.^[4]^ With regard to an favorable outcome obtained with FET, an increasing amount of reproductive centers tend to adopt the freeze-all strategy. With the advent of a freeze-all policy, exploration of the risk factors associated with pregnancy and implantation rates generated from vitrified-warmed cycles is important.

FET is currently mainly performed at the cleavage stage (days 3) or at the blastocyst stage (days 5–6). Vitrified-warmed day 3 embryo transfer is still routinely applied in the clinical setting.^[5]^ Various factors are known to affect the outcome of FET. Among the clinical factors, maternal age at oocyte retrieval, embryo quality, and the number of embryos transferred could independently affect the clinical outcomes.^[6]^ Furthermore, the duration of post-thaw culture could be involved.^[7,8]^ Existing data concerning the effect of culture duration on outcomes are conflicting. Rato et al^[7]^ found higher implantation and live birth rates after a short post-thaw culture period compared with overnight culture. However, other studies showed no differences in these variables between the short culture and long culture groups.^[8,9]^ Nonetheless, these studies mainly focused on comparison between short culture and long culture, without analyzing the effect of different periods of a short duration of culture on the outcomes of FET. In a short culture, embryos are evaluated immediately after thawing and are transferred on the same day of thawing if ≥ 50% intact blastomeres were observed. Whether immediate transfer after thawing (culture time < 2 hours) affects the success rate and whether transfer after a certain time of culture can improve the outcome remain inconclusive. Without considering the duration of culture, the relative effect of embryos transferred at any time after thawing on clinical outcomes has not been evaluated.

The present study aimed to evaluate the importance of the duration of post-thaw culture in FET and identify which other characteristics in FET affect the clinical outcome after FET. Specifically, the association of short-term culture duration and clinical outcomes after FET were analyzed.

## 2 Materials and methods

### 2.1 Study design

This study was approved by the institutional ethical review board of Sir Run Run Shaw Hospital, The Affiliated Hospital of the College of Medicine, Zhejiang University. In this retrospective cohort study, all women who underwent first vitrified-warmed day 3 embryo transfer treatment between January 2012 and November 2017 were included for analysis. Those cycles with missing data and without accurate medical record numbers were excluded. A total of 10,464 vitrification cycles were included in this retrospective cohort study.

### 2.2 Evaluation of embryos after vitrified-warming

Morphological survival was analyzed according to the cell stage before cryopreservation. Embryos were evaluated for morphological warm survival immediately after warming. Embryos with at least 50% of their cells intact were considered to have survived and able to be transferred. An embryo with 6to 12 equal blastomeres and ≤ 20% fragmentation was defined as good quality.

### 2.3 Cryopreservation protocols

The vitrification procedure was performed using Cryotop strip (open vitrification procedure) or CBS High Security Vitrification straws (closed vitrification procedure) in combination with ethylene glycol-dimethylsulfoxide-sucrose (Kitazato Supply Co, Fujinomiya, Japan) as the cryoprotectant.

### 2.4 Preparation of the vitrified embryo transfer (FET) cycle

In our center, 3 main types of clinical protocols were used for endometrial preparation as follows: the natural cycle, hormone replacement cycle, and human menopausal gonadotrophin (HMG)-stimulated cycle.^[10]^ For patients with regular menstrual cycles, the natural cycle was proposed and embryo transfer was performed 3 days after ovulation, or 4.5 days after the peak of luteinizing hormone. In patients with irregular ovulation or an anovulatory cycle, hormone replacement treatment or an HMG-stimulation protocol was used.

### 2.5 Categories of groups

Patients were divided into the overall group (0.5–72 hours of post-thaw culture) and the short-term culture group (0.5–8 hours of post-thaw culture). Moreover, to clarify whether the factors associated with clinical outcome were different in younger patients and older patients, we divided the patients into the 2 following groups: ≤ 35 and > 35 years, according to the patients’ ages. Additionally, to examine the results more specifically, patients were stratified into 7 groups according to the post-thaw culture time as follows: group 1, the culture time was < 2 hours; group 2, the culture time was ≥ 2 hours and < 3 hours; group 3, the culture time was ≥ 3 hours and < 4 hours; group 4, the culture time was ≥ 4 hours and < 5 hours; group 5, the culture time was ≥ 5 hours and < 6 hours; group 6, the culture time was ≥ 6 hours and < 8 hours; and group 7, the culture time was ≥ 48 hours and ≤ 72 hours.

### 2.6 Outcome measures

Biochemical pregnancy was assessed by detecting serum β-hCG levels at 12 days after transfer and β-hCG levels ≥ 50 IU/L were considered positive. The pregnancy rate was defined as the number of β-hCG-positive patients divided by transferred cycles. Five weeks after transfer, the number of gestational sacs and the presence of fetal heart motion were evaluated by ultrasound. The implantation rate was calculated using the number of observed intrauterine gestational sacs divided by the number of embryo transferred. The live birth rate was defined as the number of newborns per number of embryos transferred.

### 2.7 Statistical analysis

Binary logistic regression analysis was used to identify independent correlates between the pregnancy rate and possible confounding factors. Ordinary least square regression models were conducted to evaluate the associations of variables of interest and the implantation rate, live birth rate, and live birth weight. All statistical analyses were performed using Statistical Package for the Social Sciences Version 22.0 (SPSS Inc., Chicago IL). A *P* value of < .05 was considered to be statistically significant.

## 3 Results

**Figure 1:**
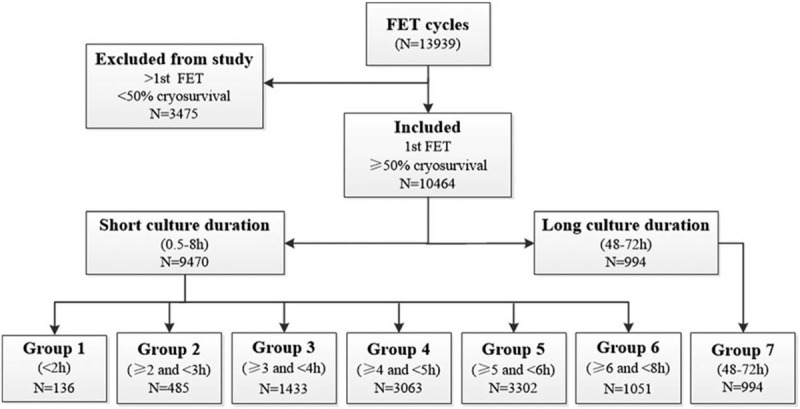
Flow chart summarizing thawed embryos accountability of the study.

Between January 2012 and November 2017, a total of 10,464, first vitrified-warmed transfer cycles were analyzed. There were 9470 cycles in the short-term culture group and 994 cycles in the 48 to 72-hours culture group (Fig. 1).

Binary logistic regression analysis was used to analyze the associations between pregnancy rates and risk factors. In the overall and short-term culture groups, after adjusting for known confounding factors such as maternal age, duration of infertility, endometrial preparation protocol, number of transferred embryos, number of transferred good quality embryos, and endometrial thickness, the pregnancy rate was positively associated with the post-thaw culture time (adjusted odds ratio [aOR] 0.986, 95% confidence interval [CI] 0.983–0.99, *P* < .001; aOR 0.944, 95% CI 0.907–0.983, *P* < .01, respectively), the number of transferred embryos (aOR 0.642, 95% CI 0.579–0.711, *P* < .001; aOR 0.624, 95% CI 0.557–0.699, *P* < .001, respectively), the number of transferred good quality embryos (aOR 0.592, 95% CI 0.555–0.631, *P* < .001; aOR 0.600, 95% CI 0.562–0.641, *P* < .001, respectively), and endometrial thickness (aOR 0.919, 95% CI 0.896–0.942, *P* < .001; aOR 0.917, 95% CI 0.893–0.942, *P* < .001, respectively) (Table 1). Maternal age was negatively associated with the pregnancy rate in the overall and short-term culture groups (aOR 1.089, 95% CI 1.079–1.100, *P* < .001; aOR 1.091, 95% CI 1.08–1.102, *P* < .001, respectively). In each age group (≤ 35 and > 35 years), the clinical pregnancy rate was significantly associated with the number of transferred embryos, the number of transferred good quality embryos, endometrial thickness, and maternal age. Interestingly, the association between the post-thaw culture time and pregnancy rate remained significant in the overall and short-term culture groups of ≤ 35 years and the overall group of > 35 years. However, in the short-term culture group of > 35 years, the pregnancy rate was not significantly associated with the post-thaw culture time with adjustment for the same factors (*P* > .05).

**Table 1:**
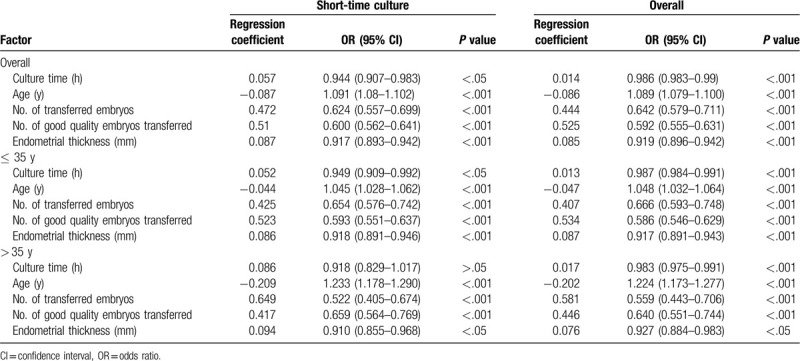
Binary logistic regression analysis between pregnancy rates and associated factors.

The implantation and live birth rates according to the duration of post-thaw culture are shown in Table 2. Ordinary least square regression analyses were performed to determine the relationships between the duration of post-thaw culture and implantation and live birth rates. Overall, even after correction for several potential confounders, positive associations were found between the duration of post-thaw culture and the implantation rate (*P* < .001) and live birth rate (*P* < .001). However, the implantation and live birth rates were not significantly associated with the duration of post-thaw culture in the short-term culture group. Additionally, maternal age and the number of transferred embryos were independent predictors of implantation and live birth rates. In subanalysis of factors associated with implantation and live birth rates in the ≤ 35 and > 35 years groups, multiple linear regression with the same factors as in the main analysis showed that the duration of post-thaw culture was not associated with implantation and live birth rates in the > 35 years group. Moreover, the duration of post-thaw culture did not affect live birth weight. The number of live births was the only independent predictor of live birth weight (Table 3).

**Table 2:**
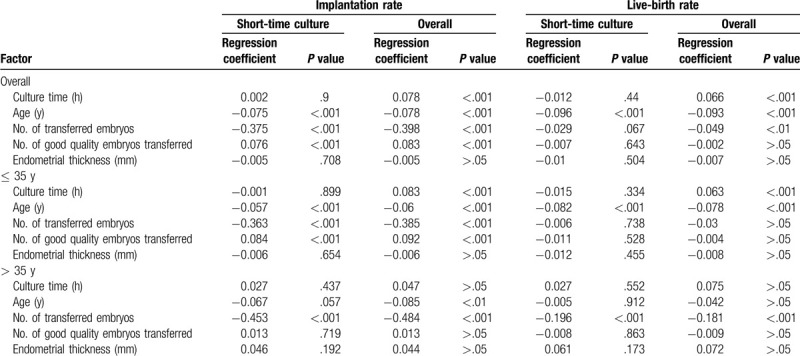
Ordinary least square regression analysis of implantation and live-birth rates.

**Table 3:**
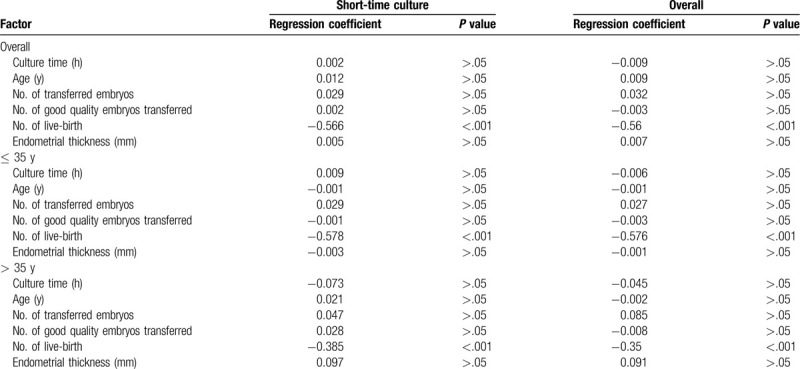
Ordinary least square regression analysis of live-birth weight.

**Figure 2:**
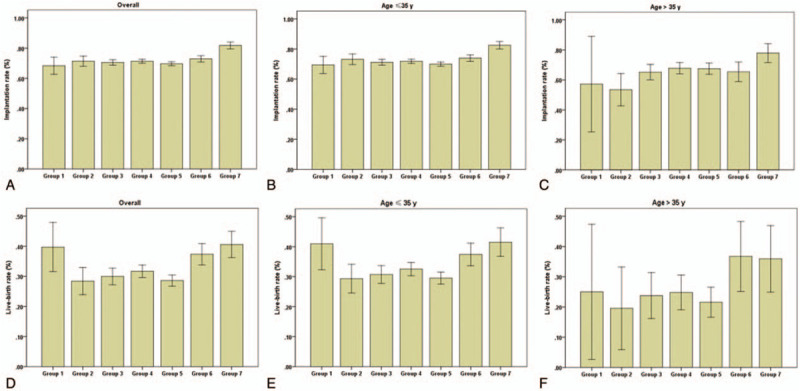
Implantation and live birth rates according to the duration of post-thaw culture. Embryos that were cultured for different periods showed distinct outcomes. The implantation and live birth rates gradually increased across the 7 groups.

To analyze the distribution of patients, patients were stratified into 7 groups according to the post-thaw culture time (Table 4). We found that the duration of post-thaw culture was 3 to 8 hours in 84.6% of patients and up to 48 to 72 hours in 9.5% of patients. The mean number of transferred embryos per cycle, transferred good embryos per cycle, and twin rates were significantly lower in group 7. No significant differences in live birth weight of singleton pregnancies and twin pregnancies were found among the 7 groups. However, despite the lower number of embryos and good embryos to be transferred in group 7, the pregnancy, implantation, and live birth rates were not decreased. Conversely, the implantation and live birth rates gradually increased across the 7 groups (Fig. 2).

**Table 4:**
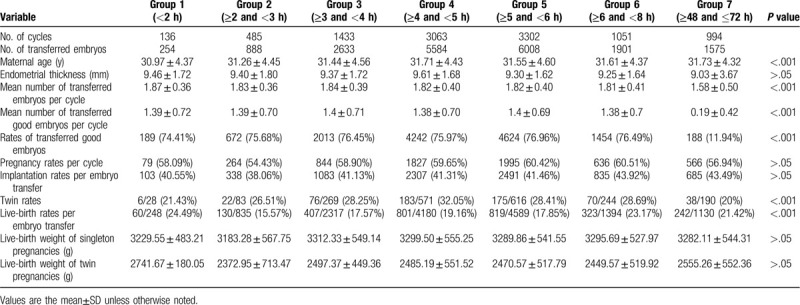
Clinical outcomes in 7 groups stratified by culture time.

## 4 Discussion

In this large cohort study, we found a significant association between the duration of post-thaw culture and pregnancy, implantation, and live birth rates. However, when embryos were transferred at the day of thawing, there was no association of post-thaw culture time with implantation and live birth rates. Moreover, the duration of post-thaw culture did not affect live birth weight.

To the best of our knowledge, this is the first large retrospective study to evaluate the association of the duration of post-thaw culture with clinical outcome after vitrified-warmed day 3 embryo transfer. There are 3 options for vitrified-warmed day 3 embryo transfer. One option is blastocyst transfer after culture for 48 to 72 hours, another is overnight culture transfer after culture for 24 hours, and the third is transfer on the day of thawing. The first 2 transfer methods may have the possibility of elimination of an undeveloped embryo, which may reduce the number of transferred embryos or cancel embryo transfer. Therefore, compared with the third method, the first 2 methods can improve the pregnancy rate because of elimination of embryos without developmental potential.^[11,12]^ Furthermore, embryos that are not cleaved after overnight culture result in low pregnancy rates. However, a previous study reported that FET with or without overnight culture after thawing did not have a different pregnancy rate.^[8]^ To some extent, short-term culture is even better than overnight culture for the implantation and live birth rates.^[7]^ Therefore, environmental factors may have an important role in embryo implantation and developmental potential during overnight culture. In our center, vitrified-warmed embryos were mainly transferred on the same day of warming (90.5%). Only a small number of day 3 embryos (9.5%) were transferred after 48 to 72 hours of culture and no embryos were transferred after overnight culture. In the current study, the overall pregnancy rate of vitrified-warmed day 3 embryo transfer after short-term culture was 59.6% (5645/9470), which is in line with previous studies.^[13]^ Our results reinforce the accuracy and advantages of a frozen-thawed embryo selection approach strictly based on cryosurvival of blastomeres. In all 9470 cycles of day 3 embryos transferred on the day of thawing, the duration of post-thaw culture ranged from 0.5 to 8 hours. We found that the duration of post-thaw culture had a positive correlation with the pregnancy rate, but had no significant correlation with embryo implantation and live birth rates. However, in group analysis, except for group 1, we found an increasing trend of embryo implantation and live birth rates in the groups. The live birth rate in group 1 was higher than that in the other groups. Furthermore, the age of patients in group 1 was younger than that in the other groups. Additionally, the mean number of transferred good embryos per cycle was relatively high, which may have led to higher live birth rates in group 1. Moreover, the present study showed that, except for the duration of culture, maternal age was an independent predictor of pregnancy, implantation, and live birth rates. Therefore, we divided the patients into 2 groups of ≤ 35 and > 35 years, according to the patients’ age. We found that there was a positive correlation between the duration of culture and pregnancy rate in the younger age group, but no correlation in the advanced age group. This finding suggested that appropriately prolonging the duration of culture could improve the pregnancy rate when vitrified-warmed day 3 embryos were transferred on the day of warming.

We also wanted to examine whether the duration of post-thaw culture is associated with the live birth weight. Therefore, we analyzed the factors associated with live birth weight. We found that the duration of post-thaw culture did not affect live birth weight and this result could not be overcome by prolonging the in vitro culture time. In contrast to our findings, Zhang et al^[1]^ showed that the culture period had a significant and strong effect on birth weight of singleton newborns. Therefore, the live birth weight increases with a longer culture period, However, a recent study by de Vos et al^[14]^ reported a significantly lower birth weight in singletons born after vitrified-warmed blastocyst transfer compared with cleavage-stage transfer. In the present study, we did not find a correlation between the duration of culture and fetal weight, which may be because the culture durations are much the same. Although the difference in duration of post-thaw culture was nearly 8 hours, this was still not long enough to affect the live birth weight. Moreover, the present study showed that even though frozen-thawed day 3 embryos were cultured to the blastocyst stage, the live birth weight did not increase or decrease. These contradictory findings could be largely attributed to the limited population size of live birth obtained after blastocyst transfer. Furthermore, possible factors known to affect intrauterine fetal growth, such as pregnancy-induced hypertension, gestational diabetes, and preeclampsia, were not excluded from our study.

The design of the current study may have resulted in some biases that were inherent because of its retrospective nature, which might have affected our results. Therefore, we meticulously screened the data with strict criteria and without a single case lost to follow-up. One of the main strengths of our study is the large sample size, including 10,464 cycles, with first vitrified-warmed transfer cycles within a single institution. This provided useful information on the rates of pregnancy, implantation, and live birth. Most importantly, in the present study, laboratory conditions, the embryo selection criteria for cryopreservation, and procedures, as well as culture medium, remained consistent during the study period.

In conclusion, our study shows that the pregnancy rate is positively associated with the duration of post-thaw culture. The probability of pregnancy becomes greater with a longer duration of post-thaw culture. Therefore, under the condition of not affecting work shifts, properly prolonging the duration of post-thaw culture to improve the outcome of FET should be considered. Large prospective studies should be conducted to confirm this finding in the future.

## Acknowledgments

The authors sincerely thank other investigators and physicians who made contributions to this study. The authors thank Ellen Knapp, PhD, from Liwen Bianji, Edanz Group China (www.liwenbianji.cn/ac), for editing the English text of a draft of this manuscript.

## Author contributions

**Analysis:** Yamei Xue.

**Data curation:** Haiyan Zhu, Wen Xu, Xiaoying Jin.

**Formal analysis:** Xiaoying Jin.

**Funding acquisition:** Wen Xu.

**Investigation:** Xiaomei Tong.

**Project administration:** Songying Zhang.

**Software:** Yamei Xue.

**Supervision:** Songying Zhang.

**Writing – original draft:** Haiyan Zhu.

**Writing – review & editing:** Haiyan Zhu.
